# Supplementary material for: Low variability of the Atlantic Meridional Overturning Circulation throughout the Holocene
Source: Nat Commun. 2025 Jul 22;16:6748. doi: 10.1038/s41467-025-61793-z (PMC12284099; doi:10.1038/s41467-025-61793-z)
Supplement: Supplementary file 1 — Supplementary Information [file 41467_2025_61793_MOESM1_ESM.pdf]

Supporting information

Discussion

bOpal influence on $^{231}Pa/^{230}Th$

Protactinium is more efficiently scavenged by biogenic Opal (bOpal) compared to most other particle types [1-3].Therefore, with increasing bOpal fluxes higher $^{231}Pa/^{230}Th$ ratios are expected. However, in most of the Atlantic's seafloor sediments linear correlations of bOpal content with $^{231}Pa/^{230}Th$ are not observed [4]. This might be due to changes in remineralization and preservation rates of bOpal particles [5], the presence of other particles with high adsorption properties for ^{231}Pa [6], and the role of AMOC preferentially advecting ^{231}Pa [7], dominating the sedimentary $^{231}Pa/^{230}Th$ over the effect of opal-scavenging for most of the Atlantic Ocean.

A basin-wide comparison of sedimentary bOpal concentrations with $^{231}Pa/^{230}Th$ over the last 120 ka, indicates little (bOpal <~10%) to no (bOpal <~5%) correlation between both parameters (Supplementary Fig. 1). Across the five core locations investigated here, bOpal concentrations remain consistently below ~7% (Supp. Fig. 2). This is notably below the empirically observed threshold displayed in Supp. Fig. 1, wherein a linear relationship between bOpal and $^{231}Pa/^{230}Th$ is observed only for concentrations above ~10% [8]. Overall, the five down-core records show no correlation between the $^{231}Pa/^{230}Th$ ratios and bOpal concentration, with p-values far above 0.05 (Supplementary Fig. 2) [9].

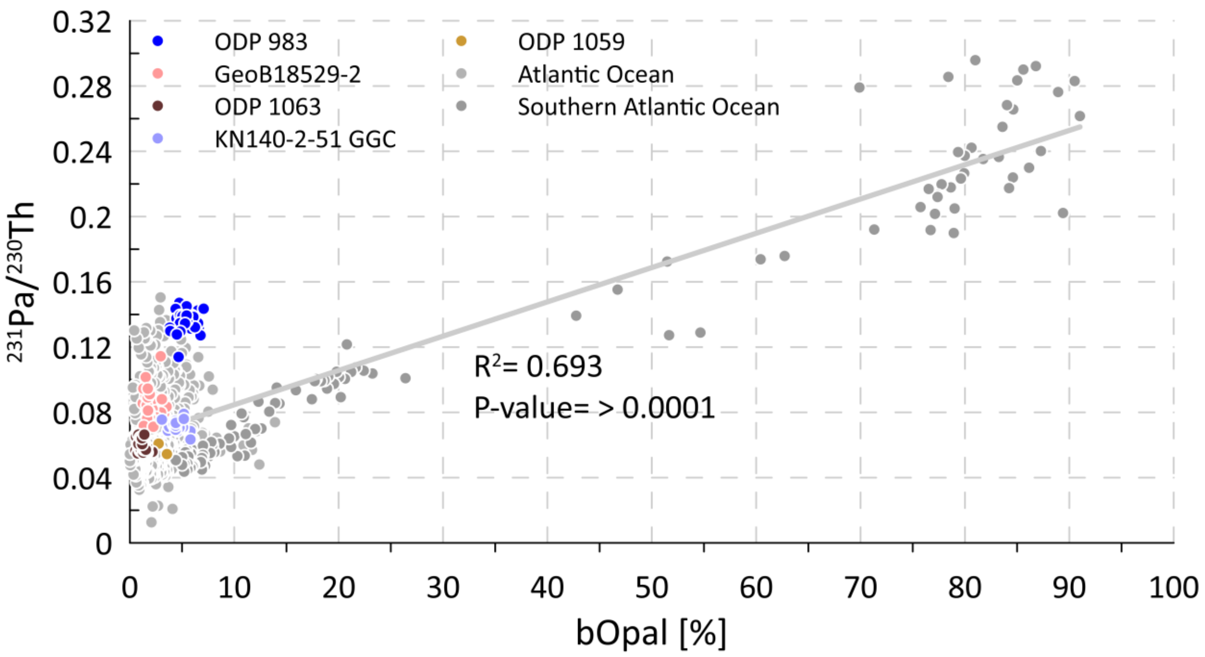

Supplementary Fig. 1: Cross-plot of the biogenic Opal (bOpal) concentration vs. $^{231}Pa/^{230}Th$ ratios over the last 120 ka BP from the entire Atlantic Ocean. *The colored data points indicate the bOpal and $^{231}Pa/^{230}Th$ ratios of the Holocene from this study. Darker grey points indicate data from the Southern Atlantic Ocean.*

Despite the high $^{231}Pa/^{230}Th$ values of ODP 983 ($^{231}Pa/^{230}Th_{mean}$ = 0.137), the bOpal values range between 4 to 7% (Supplementary Fig. 2a) and are only slightly higher than bOpal concentrations of the other locations, such as KN140-2-51GGC (bOpal = 4 to 6%) with distinctly lower $^{231}Pa/^{230}Th$ ratios ($^{231}Pa/^{230}Th_{mean}$ = 0.071; Supp. Fig. 2d). In contrast, for high opal regions, such as the Southern Ocean, a nearly linear relationship between both parameters has been found (Supp. Fig. 1). Following this, bOpal concentrations of around 40% would be expected for causing ODP 983's high $^{231}Pa/^{230}Th$ ratios, by far exceeding the production ratio ($^{231}Pa/^{230}Th_{mean}$ = 0.137). Thus, bOpal most likely cannot solely account for such high $^{231}Pa/^{230}Th$ values.

For all five $^{231}Pa/^{230}Th$ profiles we thus conclude that bOpal is not the main driver of the temporal evolution of the respective $^{231}Pa/^{230}Th$ profiles and other factors such as location, AMOC, or BNL-intensity exert a more important role on the resulting $^{231}Pa/^{230}Th$ ratios.

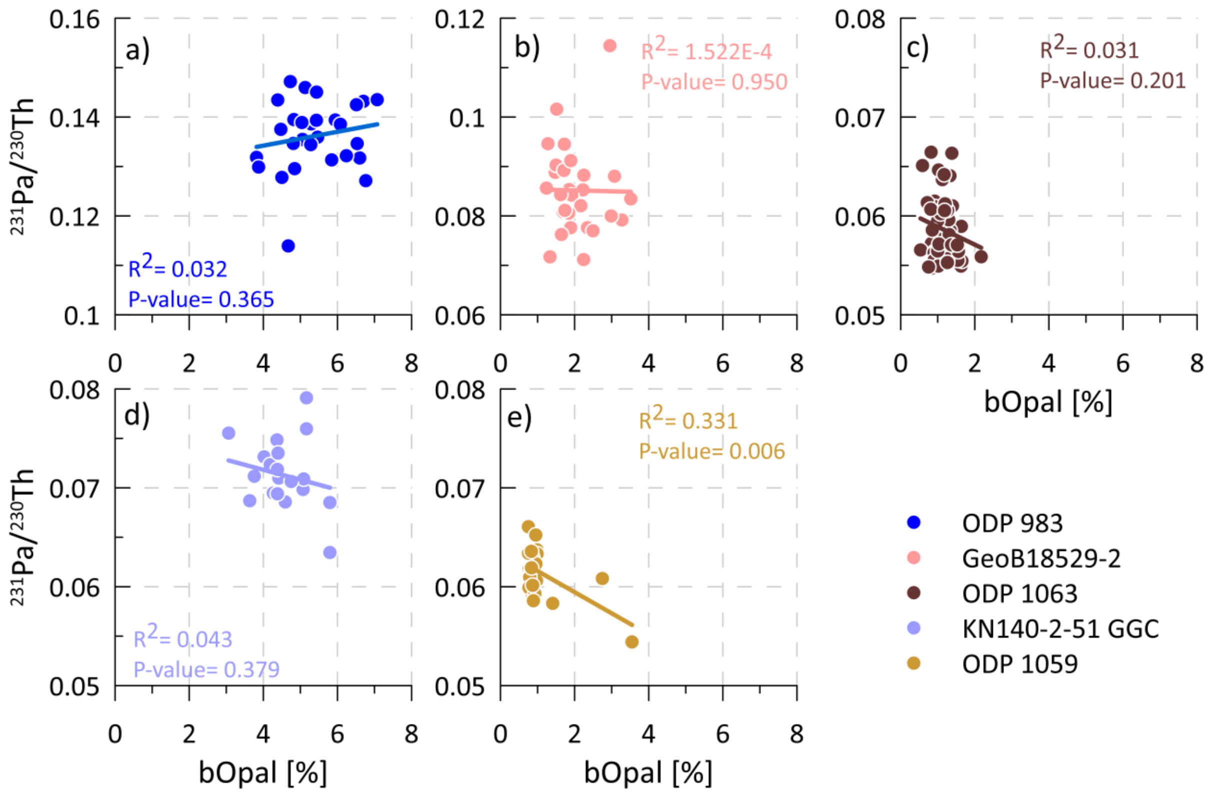

Supplementary Fig. 2: Cross-plots of Holocene $^{231}Pa/^{230}Th$ versus biogenic Opal (bOpal) concentrations for the five North Atlantic cores investigated in this study. *Additionally, given are the p-value and R^2 of every bOpal - $^{231}Pa/^{230}Th$ relation.*

Holocene AMOC strength

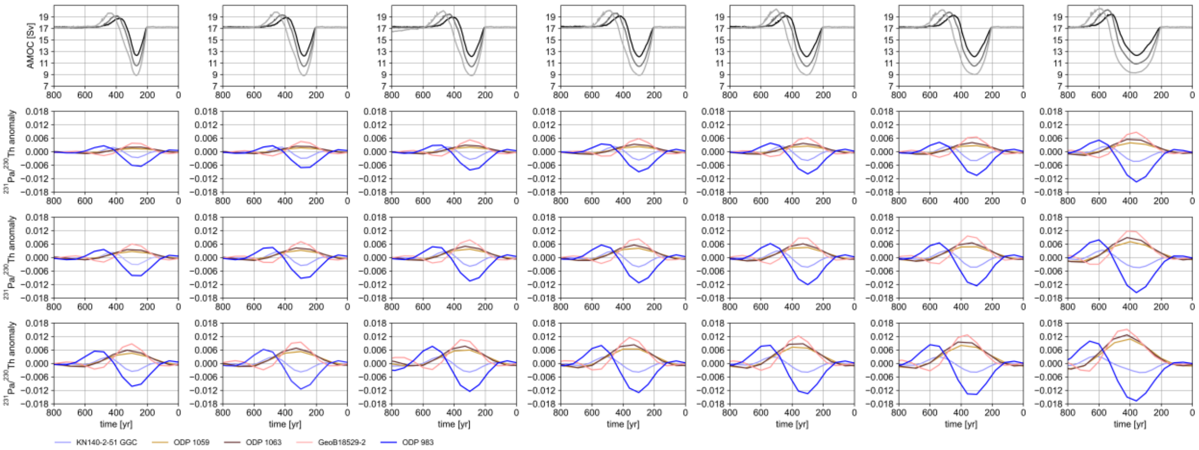

Supplementary Fig. 3: Simulated AMOC perturbations. *Various idealized scenarios with different durations of perturbations (100, 120, 140, 160, 180, 200 and 300 years) and magnitudes of potential AMOC weakening (-4.9, -6.8, -8.5 Sv) at the respective core sites of this study and the resulting* $^{231}Pa/^{230}Th$ *signals. Site-specific factors like sedimentation rate (Supplementary Tbl. 1), water depth (Supplementary Tbl. 1), sampling interval (1cm) and bioturbation (5cm depth) were considered of each location for these simulations.*

Early Holocene and 8.2 ka event

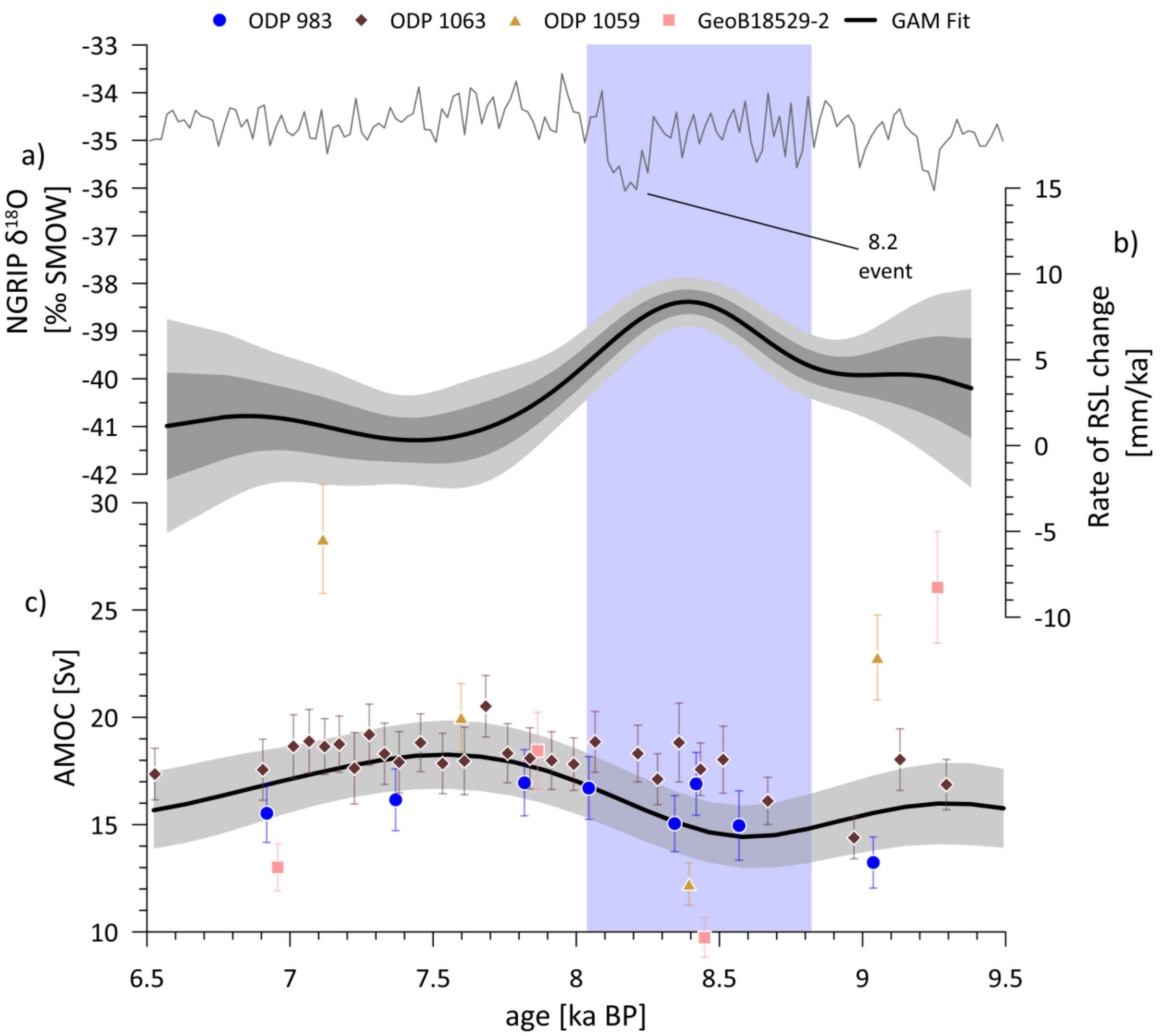

Supplementary Fig. 4: Atlantic Meridional Overturning Circulation (AMOC) strength during melt-water events in the early Holocene. *(a) NGRIP (North Greenland Ice Core Project) $\delta^{18}O$ [10, 11] (b) Rate of relative sea level change (RSL), with 1σ and 2 σ confidence limits [12]. (c) GAM (Generalized Additive Model) fit of the Holocene AMOC strength (with its 95% confidence interval) calculated from the Bern3D quadratic relationships between measured $^{231}Pa/^{230}Th$ and AMOC flux of each core location (colored data points, including standard errors) [13]. The time periods around the strongest sea level rise was highlighted with the blue vertical bar.*

Materials and Methods

Supplementary Table 1: Overview of the sediment cores considered in this study and their respective data. *To get the mean temporal resolution of the Bermuda Rise record the number of samples of OCE326-GGC5 and ODP 1063 were combined. The list of the respective records can be found in the Supplementary data sheet.*

**Core**	**Latitude**	**Longitude**	**Water Depth (m)**	**Mean sedimentation rate (ka/cm)**	**Number of $^{231}Pa/^{230}Th$ data (of this study)**	**Mean temporal resolution (sample per ka)**	**$^{231}Pa/^{230}Th$ data reference**	**Age model reference**
ODP 983	60.40	-23.64	1984	15.54	33 (33)	2.8	This study	[14, 15]
GeoB18529-2	41.88	-47.56	3989	16.33	28 (28)	2.4	This study	This study
OCE326-GGC5	33.70	-57.60	4550	8.9	14		[16]	[16]
ODP 1063	33.69	-57.61	4584	10.97	86 (17)	7.4	[17]; this study	This study
KN140-2-51GGC	32.78	-76.28	1790	22.7	39	3.3	[18]	[18]
ODP 1059	31.67	-75.42	2985	9.79	33 (25)	2.8	[19] , this study	This study

Age Models

Supplementary Table 2: Overview of the uncalibrated and calibrated (calib.) radiocarbon ages considered in this study [20]. *Ages are always given in ka. The errors of the uncalibrated radiocarbon ages are given in 1 sigma standard deviation. More information of the radiocarbon ages can be found in the supplementary data sheet.*

Core	Section	Mean depth [cm]	Lab ID	^{14}C age	^{14}C age sd [±]	Calib. age [median]	Calib. age min. [95%]	Calib. age max. [95%]	Included in Age model	Reference
GeoB18529-2		2	418847	1.25	0.03	0.69	0.53	0.87	included	This study
GeoB18529-2		50	418848	4.51	0.03	4.56	4.34	4.81	included	This study
GeoB18529-2		114	BE-12549.1.1	8.70	0.05	9.22	8.99	9.44	included	This study
GeoB18529-2		188	BE-12550.1.1	10.24	0.06	11.25	11.01	11.59	included	This study
GeoB18529-2		264	BE-12551.1.1	13.20	0.05	15.09	14.81	15.39	included	This study
GeoB18529-2		282	BE-12552.1.1	14.38	0.05	16.63	16.33	16.93	included	This study
GeoB18529-2		300	418849	16.52	0.05	19.08	18.82	19.38	included	This study
ODP1059	A1H1W	30	BE-19464.av	3.61	0.23	3.58	3.23	3.93	included	This study
ODP1059	A1H1W	53	BE-19462.1.1	5.28	0.03	5.65	5.32	5.95	included	This study
ODP1059	B1H1W	22.5	BE-16592.1.1	2.98	0.04	2.82	2.44	3.18	included	This study
ODP1059	B1H1W	26.5	BE-16593.1.1	3.42	0.05	3.34	2.96	3.71	included	This study
ODP1059	B1H1W	32.5	BE-16594.1.1	3.86	0.04	3.89	3.51	4.28	included	This study
ODP1059	B1H1W	42.5	BE-19466.1.1	4.82	0.03	5.13	4.79	5.47	included	This study
ODP1059	B1H1W	82.5	BE-19463.av	7.81	0.07	8.29	7.95	8.63	included	This study
ODP1059	B1H1W	117	BE-19465.1.1	12.86	0.15	14.77	14.09	15.35	included	This study
ODP 1063	A1H1W	17.5	74957.1.1	1.27	0.04	0.76	0.53	0.99	included	[17]
ODP 1063	A1H1W	33.5	74958.1.1	2.22	0.05	1.75	1.46	2.06	included	[17]
ODP 1063	A1H1W	42.5	81619.1.1	2.77	0.06	2.44	2.12	2.74	included	[17]
ODP 1063	D1H1W	6.5	72008.1.1	1.05	0.05	0.57	0.33	0.78	included	[17]
ODP 1063	D1H1W	16.5	81620.1.1	1.20	0.06	0.69	0.48	0.93	included	[17]
ODP 1063	D1H1W	40.5	72009.1.1	3.12	0.05	2.87	2.56	3.19	included	[17]
ODP 1063	D1H1W	49.5	BE-16595.1.1	3.69	0.04	3.55	3.26	3.85	included	This study
ODP 1063	D1H1W	55.5	81621.1.1	3.73	0.07	3.61	3.28	3.96	not included	[17]
ODP 1063	D1H1W	57.5	BE-16597.1.1	4.22	0.05	4.25	3.91	4.57	included	This study
ODP 1063	D1H1W	64.5	BE-16598.1.1	4.88	0.04	5.09	4.81	5.40	included	This study
ODP 1063	D1H1W	76.5	72010.1.1	5.67	0.06	5.97	5.69	6.26	included	[17]
ODP 1063	D1H1W	80.5	85099.1.1	6.67	0.07	7.07	6.76	7.35	included	[17]
ODP 1063	D1H1W	84.5	85100.1.1	6.93	0.07	7.33	7.04	7.58	included	[17]
ODP 1063	D1H1W	92.5	85101.1.1	8.16	0.07	8.59	8.30	8.97	included	[17]
ODP 1063	D1H1W	97.5	85102.1.1	8.72	0.08	9.30	8.97	9.61	included	[17]
ODP 1063	D1H1W	105.5	85103.1.1	9.89	0.07	10.85	10.48	11.20	included	[17]
ODP 1063	D1H1W	112.5	72012.1.1	10.00	0.09	10.98	10.57	11.38	included	This study
ODP 1063	D1H1W	118.5	BE-16602.1.1	10.95	0.05	12.37	12.01	12.66	included	This study

Supplementary Table 3: Overview of the used rbacon input parameters for calculating the age models. *For the age modeling, mostly default rbacon settings have been used. The values of delta.R and d.STD represent the weighted mean and standard deviation, respectively, of the ten geographically closest reservoir corrections (ΔR) to GeoB18529-2 [21-23], ODP 1059 A & B [24, 25], and ODP 1063 A & D [21, 23, 26], as listed in the Marine20 database [27].*

rbacon arguments	Explanation	GeoB18529-2 (value)	ODP 1063 A (value)	ODP 1063 D (value)	ODP 1059 A (value)	ODP 1059 B (value)
**d.min**	Min. depth of age-depth model [cm]	0	3	0	10	22
**d.max**	Max. depth of age-depth model [cm]	300	47	120	53	117
**d.by**	Step size of depth [cm]	0.5	0.5	0.5	0.5	0.5
**acc.mean**	Mean value of accumulation rate [years/cm]	50	50	100	100	100
**acc.shape**	Shape parameter for accumulation rate	1.5	1.5	1.5	1.5	1.5
**mem.mean**	Mean of memory	0.5	0.5	0.5	0.5	0.5
**mem.strength**	Strength of memory	10	10	10	10	10
**cc**	Calibration curve<br>cc1= IntCal20<br>cc2= Marine20<br>cc3= SHCal20	cc2	cc2	cc2	cc2	cc2
**normal**	Radiocarbon error distribution<br>TRUE= t-distribution<br>FALSE= normal/Gaussian distribution	TRUE	TRUE	TRUE	TRUE	TRUE
**t.a**	t-distribution degree of freedom	3	3	3	3	3
**t.b**	t-distribution variance	4	4	4	4	4
**delta.R**	Reservoir correction [^{14}C years]	-41	-92	-92	-186	-186
**d.STD**	Uncertainty of the reservoir correction [^{14}C years]	63	100	100	128	128
**ssize**	Number of posterior samples for summary	4000	4000	4000	4000	4000
**prob**	Confidence level	95%	95%	95%	95%	95%

ODP 983

We applied the previously published age model of ODP 983 to our results [14, 15].

GeoB18529-2

The new age model of core GeoB18529-2 is based on seven ^{14}C ages, covering a depth range of 0.02 to 3 meter below seafloor (mbsf) (Supplementary Table 2). Details on the ^{14}C calibration are provided in the methods part of the manuscript. The ages of all 28 $^{231}Pa/^{230}Th$ data points (0.05 to 1.685 mbsf) were derived from this age model (Supplementary Fig. 5).

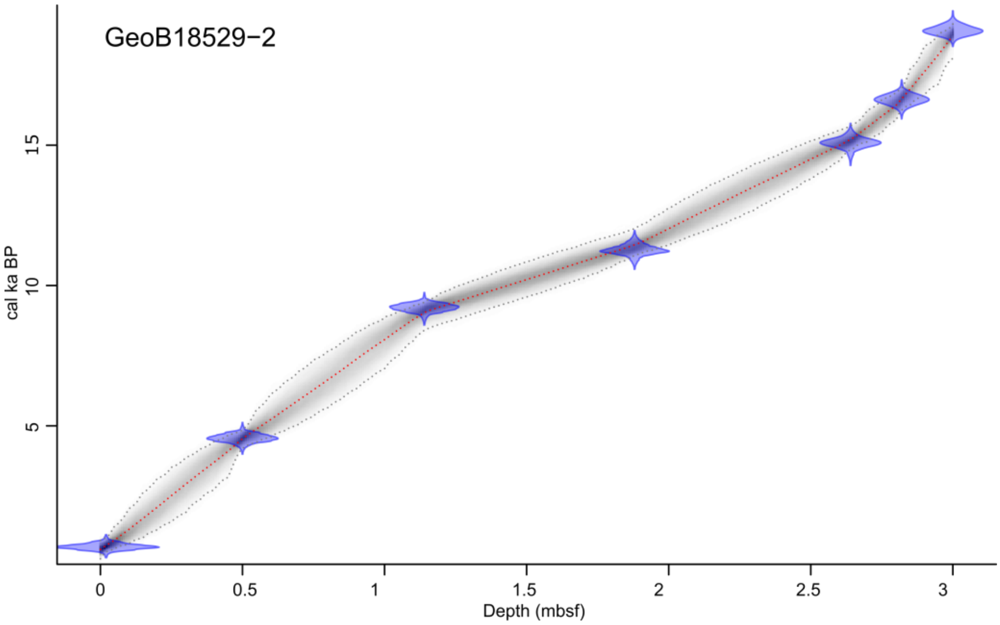

Supplementary Fig. 5: Age-depth profile of site GeoB18529-2. *This age model was constructed using calibrated radiocarbon ages (Supplementary Table 2) and their associated probability density distribution (blue shaded areas). The red dotted line represents the median age-depth relationship, with the 95% probability intervals (gray shaded areas delimited by gray dotted lines) derived using rbacon v3.3.1 [28] with the model parameters listed in Supplementary Table 3.*

ODP 1063

Samples from two holes, ODP 1063A and 1063D, were considered in this study. The existing age models for both holes were recalculated and updated [17].

For the revised age model of ODP 1063D, 15 calibrated ^{14}C ages were considered (Supplementary Table 3), covering the entire Holocene and a depth range of 0.065 to 1.185 mbsf (Supplementary Fig. 6). One radiocarbon age (lab ID: 81621.1.1) caused an irregular inflection in the age-depth relationship, resulting in an artificial spike in sedimentation rate. This radiocarbon age was therefore treated as an outlier and excluded from the construction of this age model (Supplementary Table 2).

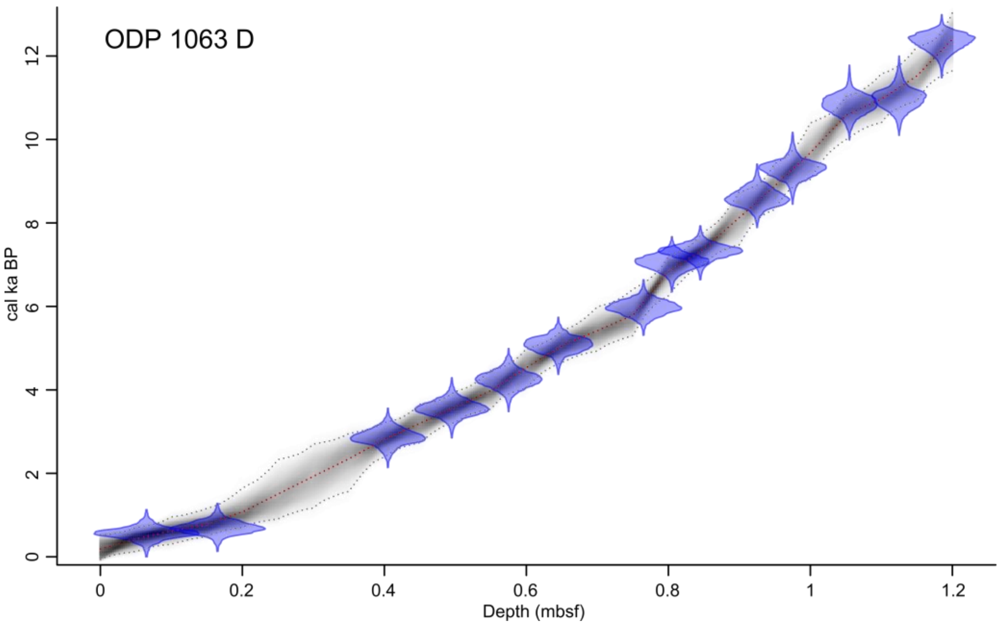

Supplementary Fig. 6: Age-depth profile of site ODP 1063D. *This age model was constructed using calibrated radiocarbon ages (Supplementary Table 2) and their associated probability density distribution (blue shaded areas). The red dotted line represents the median age-depth relationship, with the 95% probability intervals (gray shaded areas delimited by gray dotted lines) derived using rbacon v3.3.1 [28] with the model parameters listed in Supplementary Table 3.*

For ODP 1063A, the revised age model was generated from three calibrated ^{14}C ages, ranging from a depth of 0.175 to 0.425 mbsf, covering the late Holocene. Based on this age model (Supplementary Fig. 7), we assigned the ages for 10 $^{231}Pa/^{230}Th$ data points of this hole down to a depth of 0.47 mbsf.

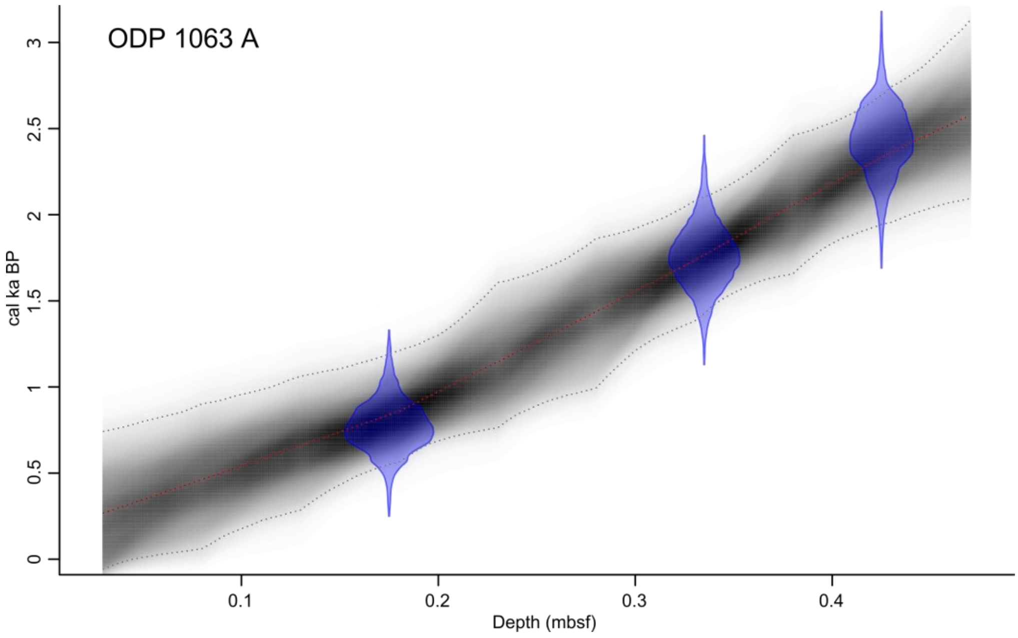

Supplementary Fig. 7: Age-depth profile of site ODP 1063A. *This age model was constructed using calibrated radiocarbon ages (Supplementary Table 2) and their associated probability density distribution (blue shaded areas). The red dotted line represents the median age-depth relationship, with the 95% probability intervals (gray shaded areas delimited by gray dotted lines) derived using rbacon v3.3.1 [28] with the model parameters listed in Supplementary Table 3.*

Additional samples from ODP 1063 A, covering a depth interval from 0.61 to 1.21 mbsf, were considered in this study. As these depths lie beyond the range of the ^{14}C-based age model for Hole A, we applied a conventional depth correlation between ODP 1063A and 1063D, following [17], using their magnetic susceptibility records [29, 30]. Based on the established tie points between both holes (Supplementary Fig. 8), we inferred the ages of the ODP 1063A data points using the age model of ODP 1063D.

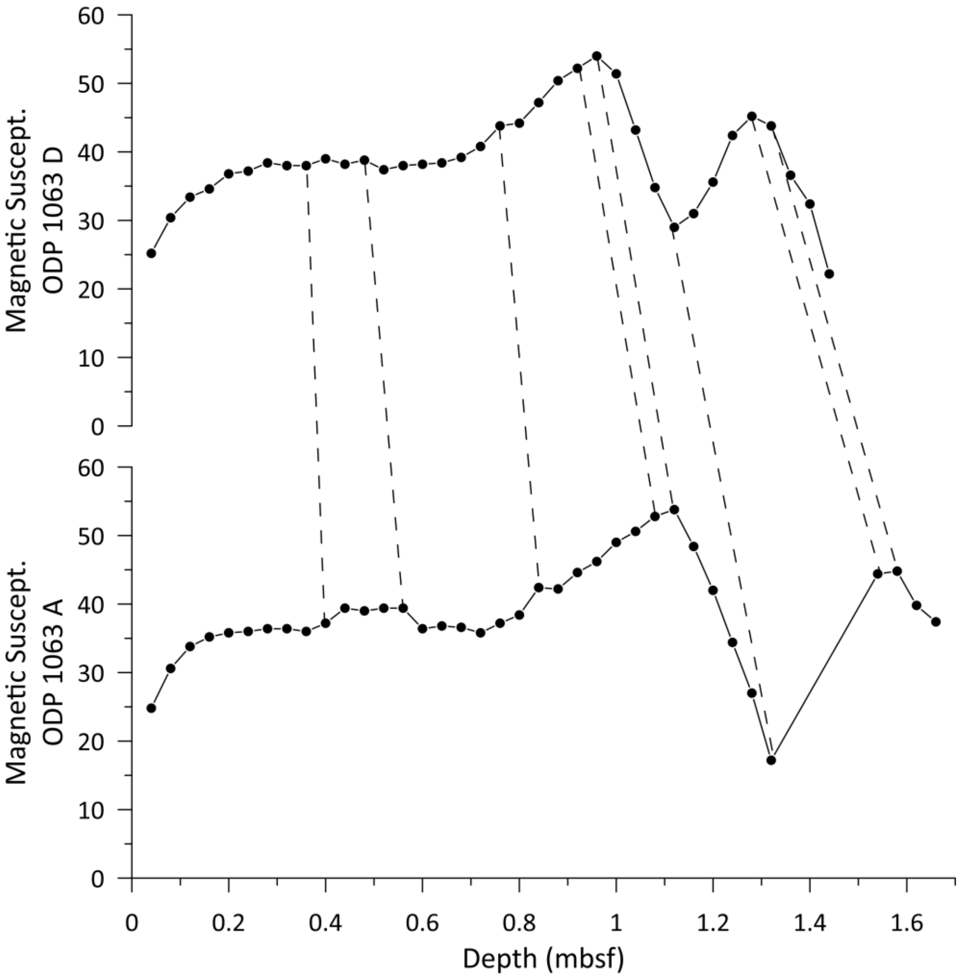

Supplementary Fig. 8: Depth correlation of ODP 1063A and D based on their magnetic susceptibility [29, 30]. *Dashed lines mark the tie points between ODP 1063A and D, following [17].*

ODP 1059

Samples from ODP Holes 1059A and 1059B were considered in this study. The revised age models of ODP 1059A and B were generated on the basis of the existing age framework [31]. For ODP 1059B, the revised age model was constructed using six calibrated ^{14}C ages (Supplementary Table 2), covering the depth range of 0.225 to 1.17 mbsf (Supplementary Fig. 9). The ages used for the $^{231}Pa/^{230}Th$ data in this study were derived from this model and span the interval from 0.225 to 0.825 mbsf. Notably, the highest uncertainty in the Bacon age-depth model occurs between ~0.9 to 1.2 to mbsf. However, none of the data points included in our analysis fall within this respective interval of increased uncertainty.

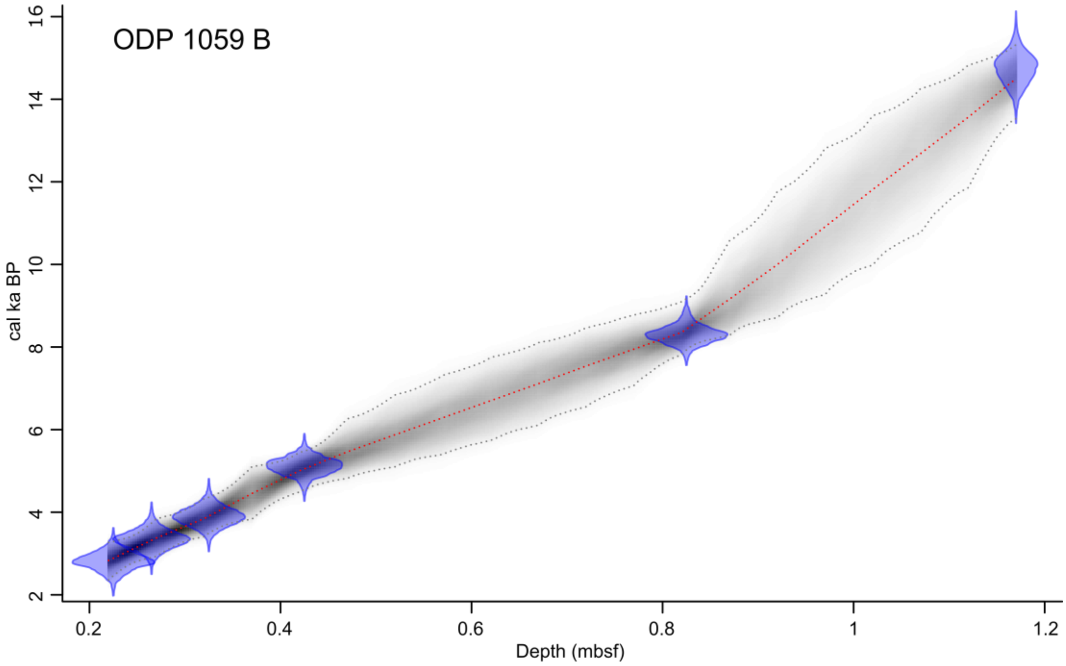

Supplementary Fig. 9: Age-depth profile of site ODP 1059B. *This age model was constructed using calibrated radiocarbon ages (Supplementary Table 2) and their associated probability density distribution (blue shaded areas). The red dotted line represents the median age-depth relationship, with the 95% probability intervals (gray shaded areas delimited by gray dotted lines) derived using rbacon v3.3.1 [28] with the model parameters listed in Supplementary Table 3.*

The revised age model of ODP 1059A was constructed using two calibrated ^{14}C ages at a depth of 0.3 and 0.53 mbsf. Based on this age model (Supplementary Fig. 10), ages were assigned to five $^{231}Pa/^{230}Th$ data points from Hole A, exiting to a depth of 0.53 mbsf.
It is worth noting, that radiocarbon age BE-19464.av has the largest uncalibrated standard deviation (230 years) of all radiocarbon ages considered in this study (Supplementary Table 2). This elevated uncertainty contributes to an increased age uncertainty in the Bacon age-depth model (Supplementary Fig. 10).

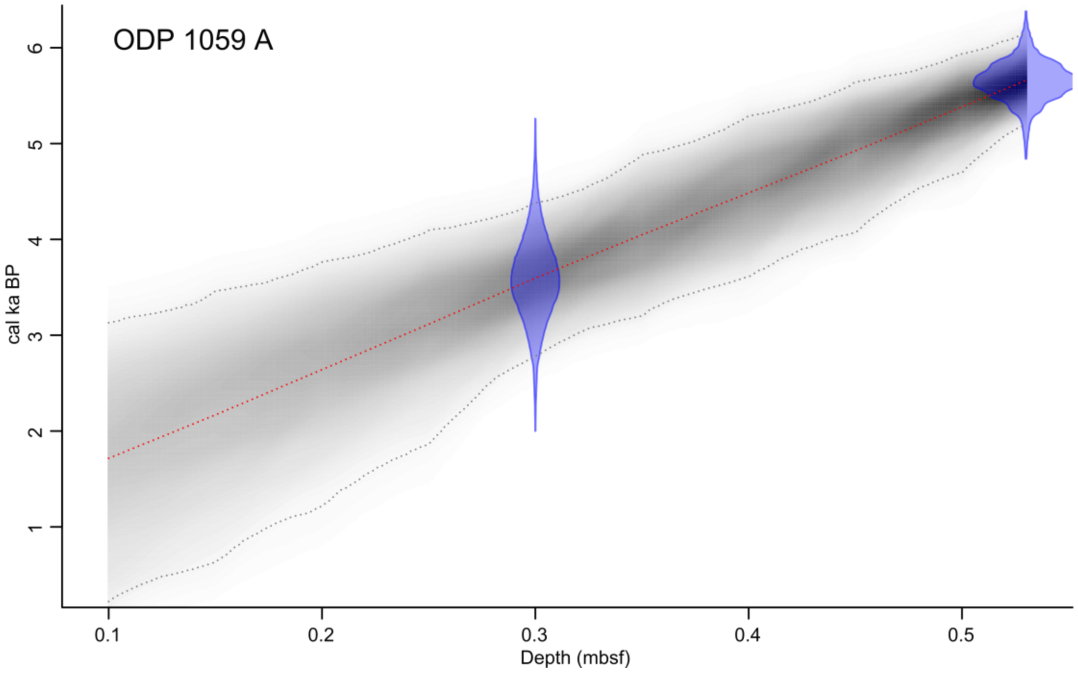

Supplementary Fig. 10: Age-depth profile of site ODP 1059A. *This age model was constructed using calibrated radiocarbon ages (Supplementary Table 2) and their associated probability density distribution (blue shaded areas). The red dotted line represents the median age-depth relationship, with the 95% probability intervals (gray shaded areas delimited by gray dotted lines) derived using rbacon v3.3.1 [28] with the model parameters listed in Supplementary Table 3.*

Five additional $^{231}Pa/^{230}Th$ samples from ODP 1059A, between 1.54 and 0.71 mbsf, were included in this study. As these depths lie beyond the range covered by the ^{14}C-based age model for ODP 1059A, a conventional depth correlation was performed between Hole ODP 1059A and 1059B, using their respective magnetic susceptibility records [32, 33]. Based on the established tie points between both holes (Supplementary Fig. 11), the ages of the ODP 1059A samples were inferred using the age model developed for ODP 1059B.

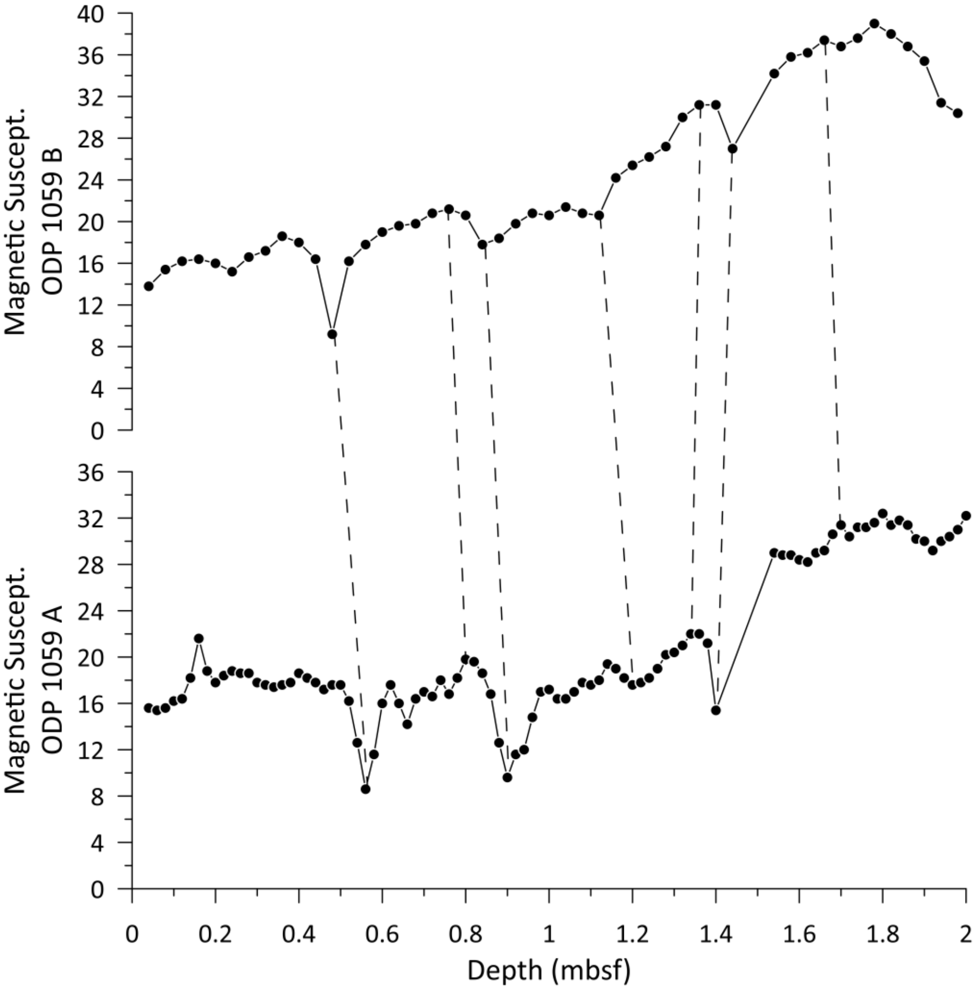

Supplementary Fig. 11: Depth correlation of ODP 1059A and B based on their magnetic susceptibility [32, 33]. *Dashed lines mark the tie points between ODP 1059 A and B.*

Bern3D

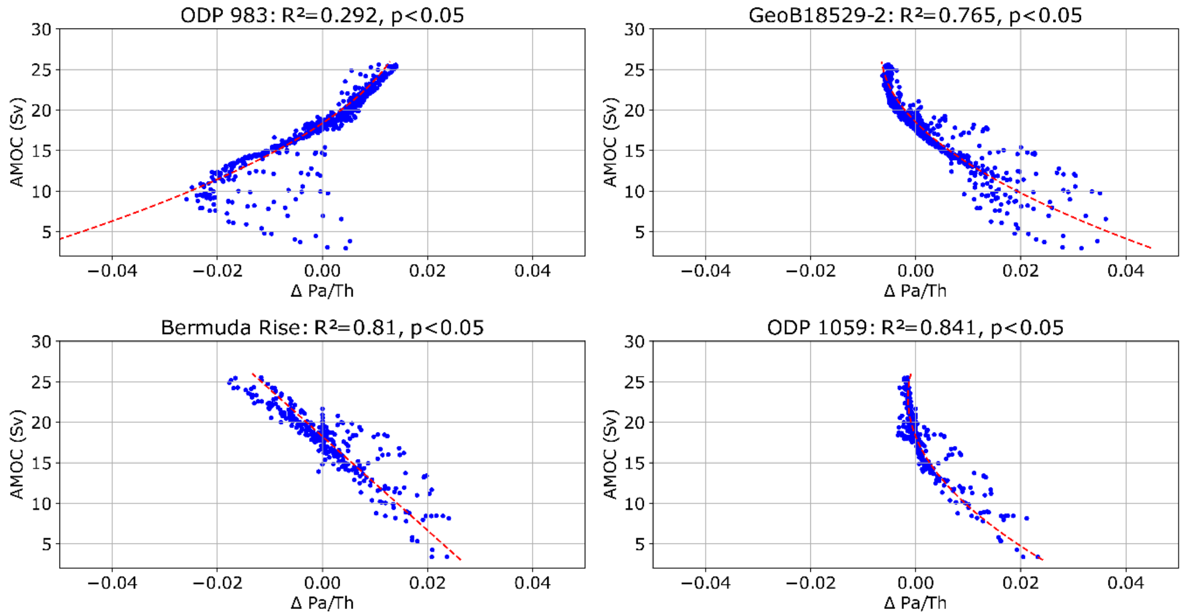

Supplementary Fig. 12: Bern3D Atlantic Meridional Overturning Circulation (AMOC)-^{231}Pa/^{230}Th relationship for the individual sites considered. *Pseudo-proxy ^{231}Pa/^{230}Th records generated from 20 idealized simulations are plotted against their respective AMOC strength (blue dots). The core-specific relationship between the modelled AMOC strength and ^{231}Pa/^{230}Th (red dashed line) is assessed with a least square's polynomial fit of degree 2. The resulting relationships are given in Supplementary Table 3.*

Supplementary Table 4: Bern3D Atlantic Meridional Overturning Circulation (AMOC)-^{231}Pa/^{230}Th relationships and RSD. *This table includes the modeled relationships and errors of Supplementary Fig. 7. x corresponds to the measured sedimentary ^{231}Pa/^{230}Th, while y gives the resulting AMOC strength in Sv. RSD= relative standard deviation.*

Core	***Bern3D AMOC-^{231}Pa/^{230}Th relationships***	***RSD (%)***
ODP 983	*$y = 3874.6x^2 + 433.1x + 18.0$*	*8.3*
GeoB18529-2	*$y = 11126x^2 + 649.7x + 18.3$*	*6.1*
ODP 1059	*$y = 23989x^2 + 1039.3x + 18.4$*	*7.6*
ODP 1063	*$y = -868.4x^2 + 446.6x + 18.0$*	*6.6*

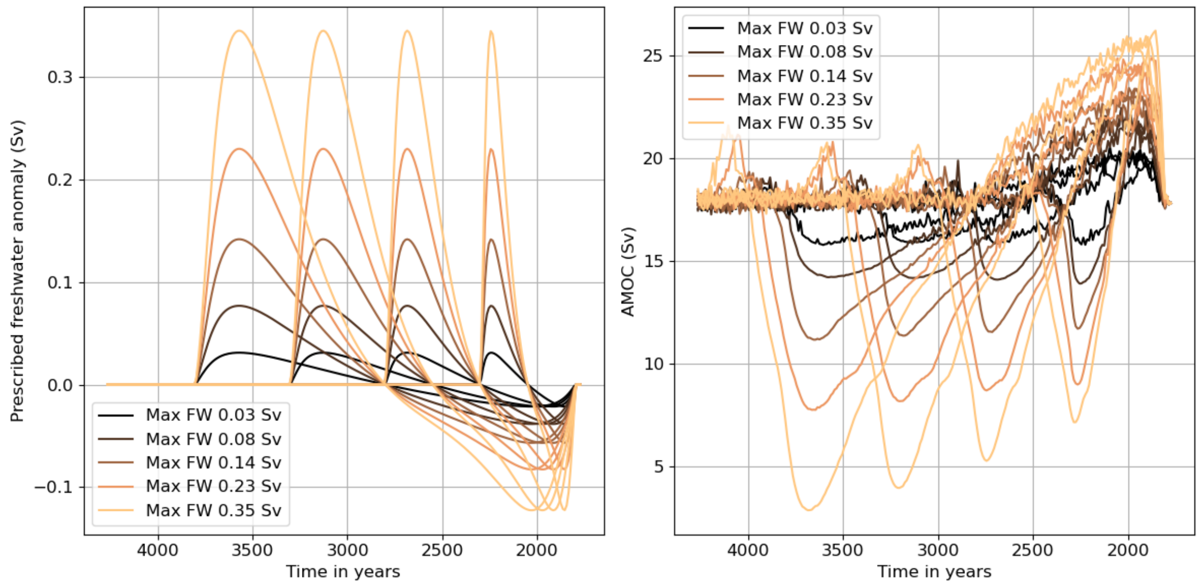

Supplementary Fig. 13: Overview of freshwater forcing and Atlantic Meridional Overturning Circulation (AMOC) strength for this set of idealized sensitivity simulations. *The freshwater perturbations were applied in a latitudinal band between 45°N and 70°N in the North Atlantic and their amplitude chosen to achieve rapid increases and gradual slowdowns of different length in AMOC strength (500, 1000, 1500, 2000 years). The different colors represent the maximum freshwater forcing, with a brighter color indicating a stronger forcing.*

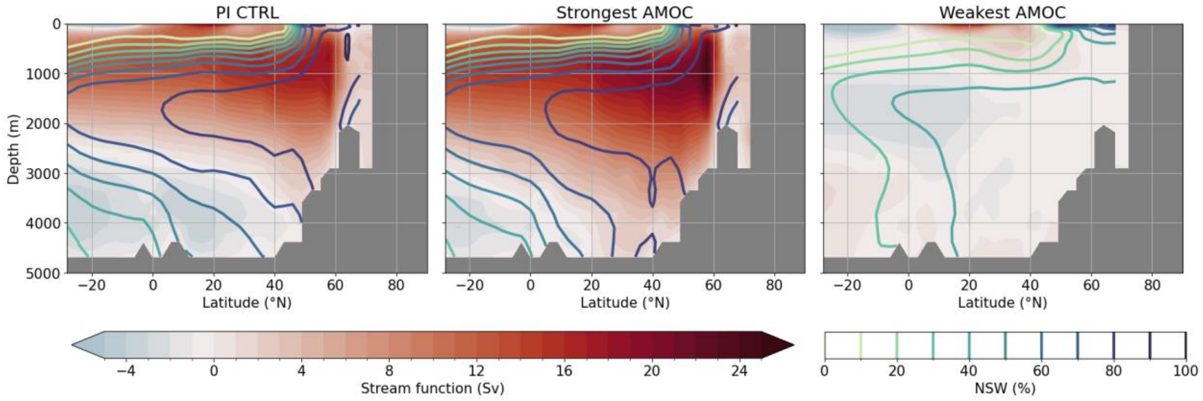

Supplementary Fig. 14: Bern3D Atlantic Meridional Overturning Circulation stream function. *Overview of the AMOC stream function (filled contours) and Northern Sourced Water concentration (contour lines) in the idealized sensitivity simulations under PI equilibrium conditions, as well as the strongest (23 Sv) and weakest (5 Sv) AMOC states. PI CTRL= Pre-industrial control.*

Supplementary references

1. Zhang, X., et al., *Adsorption of Th and Pa onto particles and the effect of organic compounds in natural seawater.* Journal of Oceanology and Limnology, 2021.
2. Chase, Z., et al., *The influence of particle composition and particle flux on scavenging of Th, Pa and Be in the ocean.* Earth and Planetary Science Letters, 2002. **204**: p. 215-229.
3. Luo, S. and T. Ku, *Reply to Comment on "On the importance of opal, carbonate, and lithogenic clays in scavenging and fractionating ^{230}Th, ^{231}Pa and ^{10}Be in the ocean".* Earth and Planetary Science Letters, 2004. **220**: p. 223-229.
4. Lippold, J., et al., *Strength and geometry of the glacial Atlantic Meridional Overturning Circulation.* Nature Geoscience, 2012. **5**(11): p. 813-816.
5. Hayes, C.T., et al., *Global Ocean Sediment Composition and Burial Flux in the Deep Sea.* Global Biogeochemical Cycles, 2021. **35**(4): p. e2020GB006769.
6. Missiaen, L., et al., *Carbon isotopes and Pa/Th response to forced circulation changes: a model perspective.* Climate of the Past, 2020. **16**(3): p. 867-883.
7. Deng, F., et al., *Evolution of ^{231}Pa and ^{230}Th in overflow waters of the North Atlantic.* Biogeosciences, 2018. **2018**: p. 1-24.
8. Lippold, J., et al., *Deep water provenance and dynamics of the (de)glacial Atlantic meridional overturning circulation.* Earth and Planetary Science Letters, 2016. **445**: p. 68-78.
9. Gerber, L., et al., *Holocene 231Pa/230Th ratios, Uranium isotopes and biogenic Opal concentrations from four North Atlantic sediment cores* 2025: PANGAEA.
10. Vinther, B.M., et al., *A synchronized dating of three Greenland ice cores throughout the Holocene.* Journal of Geophysical Research: Atmospheres, 2006. **111**(D13).
11. Rasmussen, S.O., et al., *A new Greenland ice core chronology for the last glacial termination.* Journal of Geophysical Research: Atmospheres, 2006. **111**(D6).
12. Rush, G., et al., *The magnitude and source of meltwater forcing of the 8.2 ka climate event constrained by relative sea-level data from eastern Scotland.* Quaternary Science Advances, 2023. **12**.
13. Gerber, L., et al., *Mean sub-millennial AMOC strength in the North Atlantic during the Holocene*. 2025: PANGAEA.
14. Waelbroeck, C., et al., *Consistently dated Atlantic sediment cores over the last 40 thousand years.* Scientific Data, 2019. **6**(1): p. 165.
15. Barker, S., et al., *Strengthening Atlantic Inflow Across the Mid-Pleistocene Transition.* Paleoceanography and Paleoclimatology, 2021. **36**(4).
16. McManus, J., et al., *Collapse and rapid resumption of Atlantic meridional circulation linked to deglacial climate change.* Nature, 2004. **428**: p. 834-837.
17. Lippold, J., et al., *Constraining the variability of the Atlantic Meridional Overturning Circulation during the Holocene.* Geophysical Research Letters, 2019. **46**(20): p. 11338-11346.
18. Hoffmann, S.S., J.F. McManus, and E. Swank, *Evidence for Stable Holocene Basin-Scale Overturning Circulation Despite Variable Currents Along the Deep Western Boundary of the North Atlantic Ocean.* Geophysical Research Letters, 2018. **45**(24): p. 13427-13436.
19. Süfke, F., et al., *Constraints on the northwestern Atlantic deep water circulation from ^{231}Pa/^{230}Th during the last 30,000 years.* Paleoceanography and Paleoclimatology, 2019. **34**(12): p. 1945–1958.
20. Gerber, L., et al., *14C data of three North Atlantic sediment cores during the Holocene*. 2025: PANGAEA.
21. McNeely, R., A.S. Dyke, and J.R. Southon, *Canadian Marine Reservoir Ages: Preliminary Data Assessment.* Geological Survey of Canada, 2006. **Open File 5049**(pp. 3).

22. Pearce, C., et al., *The marine reservoir age of Greenland coastal waters.* Geochronology, 2023. **5**(2): p. 451-465.
23. Campana, S.E., *Use of radiocarbon from nuclear fallout as a dated marker in the otoliths of haddock Melanogrammus aeglefinus.* Marine Ecology Progress Series, 1997. **150**: p. 49-56.
24. Thomas, D.H., *NATIVE AMERICAN LANDSCAPES OF ST. CATHERINES ISLAND, GEORGIA II. THE DATA,*. Vol. #88 2008: American Museum of Natural History.
25. Rick, T.C., et al., *Marine radiocarbon reservoir corrections (ΔR) for Chesapeake Bay and the Middle Atlantic Coast of North America.* Quaternary Research, 2017. **77**(1): p. 205-210.
26. Druffel, E.R.M., *Pulses of rapid ventilation in the north atlanic surface ocean during the past century.* Science, 1997. **275**: p. 1454-57.
27. Reimer, P.J. and R.W. Reimer, *A Marine Reservoir Correction Database and On-Line Interface.* Radiocarbon, 2016. **43**(2A): p. 461-463.
28. Blaauw, M. and J.A. Christen, *Flexible paleoclimate age-depth models using an autoregressive gamma process.* Bayesian Analysis, 2011. **6**(3).
29. Keigwin, L.D., et al., *Magnetic susceptibility on ODP Hole 172-1063A*. 2005: PANGAEA.
30. Keigwin, L.D., et al., *Magnetic susceptibility on ODP Hole 172-1063D*. 2005: PANGAEA.
31. F. Pöppelmeier, et al., *Water mass gradients of the mid-depth Southwest Atlantic during the past 25,000 years.* Earth and Planetary Science Letters, 2019. **in press**.
32. Keigwin, L.D., et al., *Magnetic susceptibility on ODP Hole 172-1059A*. 2005: PANGAEA.
33. Keigwin, L.D., et al., *Magnetic susceptibility on ODP Hole 172-1059B*. 2005: PANGAEA.
